# The global historical climate database HCLIM

**DOI:** 10.1038/s41597-022-01919-w

**Published:** 2023-01-19

**Authors:** Elin Lundstad, Yuri Brugnara, Duncan Pappert, Jérôme Kopp, Eric Samakinwa, André Hürzeler, Axel Andersson, Barbara Chimani, Richard Cornes, Gaston Demarée, Janusz Filipiak, Lydia Gates, Gemma L. Ives, Julie M. Jones, Sylvie Jourdain, Andrea Kiss, Sharon E. Nicholson, Rajmund Przybylak, Philip Jones, Daniel Rousseau, Birger Tinz, Fernando S. Rodrigo, Stefan Grab, Fernando Domínguez-Castro, Victoria Slonosky, Jason Cooper, Manola Brunet, Stefan Brönnimann

**Affiliations:** 1 Institute of Geography, University of Bern, Bern, Switzerland; 2 Oeschger Centre for Climate Change Research (OCCR), University of Bern, Bern, Switzerland; 3 Deutscher Wetterdienst (DWD), Hamburg, Germany; 4 Zentralanstalt für Meteorologie und Geodynamik (ZAMG), Vienna, Austria; 5 National Oceanography Centre, Southampton, United Kingdom; 6 Royal Meteorological Institute of Belgium, Brussels, Belgium; 7 Department of Meteorology and Climatology, Institute of Geography, University of Gdansk, Gdansk, Poland; 8 University of Sheffield, Sheffield, United Kingdom; 9 Department of Geography, University of Sheffield, Sheffield, United Kingdom; 10 Météo-France, Toulouse, France; 11 Institute of Hydraulic Engineering and Water Resources Management, Vienna University of Technology, Vienna, Austria; 12 Earth, Ocean, and Atmospheric Sciences (EOAS), Florida State University, Tallahassee, USA; 13 Department of Meteorology and Climatology, Nicolaus Copernicus University, Toruń, Poland; 14 Centre for Climate Change Research, Nicolaus Copernicus University, Toruń, Poland; 15 Climatic Research Unit (CRU), University of East-Anglia (UEA), Norwich, United Kingdom; 16 Conseil Supérieur de la Météorologie, Toulouse, France; 17 Departamento de Química y Física, Universidad de Almería, La Cañada de San Urbano, Almería, Spain; 18 School of Geography, Archaeology and Environmental Studies, University of Witwatersrand, Johannesburg, South Africa; 19 Departamento de Geografía y Ordenación del Territorio, Universidad de Zaragoza & ARAID foundation, Zaragoza, Spain; 20 Centre for Interdisciplinary Research on Montréal, McGill University, Montreal, Québec Canada; 21 National Centers for Environmental Information (NCEI), National Oceanic and Atmospheric Administration (NOAA), Asheville, NC USA; 22 Universidad Rovira i Virgili, Tarragona, Spain

**Keywords:** Palaeoclimate, Atmospheric science

## Abstract

There is a growing need for past weather and climate data to support science and decision-making. This paper describes the compilation and construction of a global multivariable (air temperature, pressure, precipitation sum, number of precipitation days) monthly instrumental climate database that encompasses a substantial body of the known early instrumental time series. The dataset contains series compiled from existing databases that start before 1890 (though continuing to the present) as well as a large amount of newly rescued data. All series underwent a quality control procedure and subdaily series were processed to monthly mean values. An inventory was compiled, and the collection was deduplicated based on coordinates and mutual correlations. The data are provided in a common format accompanied by the inventory. The collection totals 12452 meteorological records in 118 countries. The data can be used for climate reconstructions and analyses. It is the most comprehensive global monthly climate dataset for the preindustrial period so far.

## Abstract (summary)

**Table:** 

Measurement(s)	temperature of air
Technology Type(s)	weather stations
Sample Characteristic - Environment	Air - temperature, precipitation and pressre
Sample Characteristic - Location	Global Dataset

## Background & Summary

Long-term instrumental meteorological series are crucial for the understanding of interannual-to-decadal variations in climate. Analyzed together with model simulations and climate proxies they may provide new insight into underlying climate mechanisms, such as long-lasting droughts^1^, changes in atmospheric circulation^2^, or effects of volcanic eruptions^3^, and may serve as a basis for the generation of more comprehensive data products in reconstruction^4^ or data assimilation approaches^5,6^. Long-term instrumental meteorological series also serve as a reference against which human induced climate change can be compared^7^. For instance, Hawkins et al.^8^ suggested using the period 1720–1800 as a preindustrial reference, but only few records from this period are currently available. We define a record as a meteorological time series with one variable at one location.

While several global^9,10^, regional^11–13^, or national (e.g., Deutscher Wetterdienst (DWD) and the Royal Netherlands Meteorological Institute (KNMI), etc.) climate datasets exist that reach back to the 17th century, each of them has unique records and there is no comprehensive dataset across different variables. Furthermore, coverage of the early instrumental period is not as good as it could be. Brönnimann *et al*.^14^ compiled a global inventory of early instrumental meteorological measurements, showing that a large fraction of the series either have not been digitized at all or have not been integrated into global datasets. Here we follow-up on such work and present a comprehensive database of monthly early instrumental climate data.

By integrating digitally available datasets that have not so far been included in other databases and by rescuing a substantial fraction of previously non-digitalized material such as handwritten temperature logs, our database encompasses a much larger volume of early instrumental data than that from previous efforts. Although the database extends to the present, the focus is on the early instrumental periods and therefore all compiled series start before 1890, which is an appropriate year to distinguish early instrumental meteorological observations from more recent meteorological measurements in a global context^14^. In total, our database (hereafter HCLIM) comprises 12452 monthly time series of meteorological variables such as temperature (mean, maximum and minimum), air pressure, precipitation and number of wet days compiled from over 28 different source datasets complemented with 1525 newly digitized records.

All data were reformatted to a common format (SEF, described in the Format section under Data Records). Monthly means were formed from daily or subdaily data. All series then underwent a quality control (QC), followed by removing duplicates. The data are presented as monthly files in the database^15^.

The paper is structured as follows: Section 2 describes the method: compilation, data rescue, and processing; Section 3 summarizes the HCLIM database; Section 4 presents a technical validation and Section 5 provides the usage notes.

## Methods

**Fig. 1:**
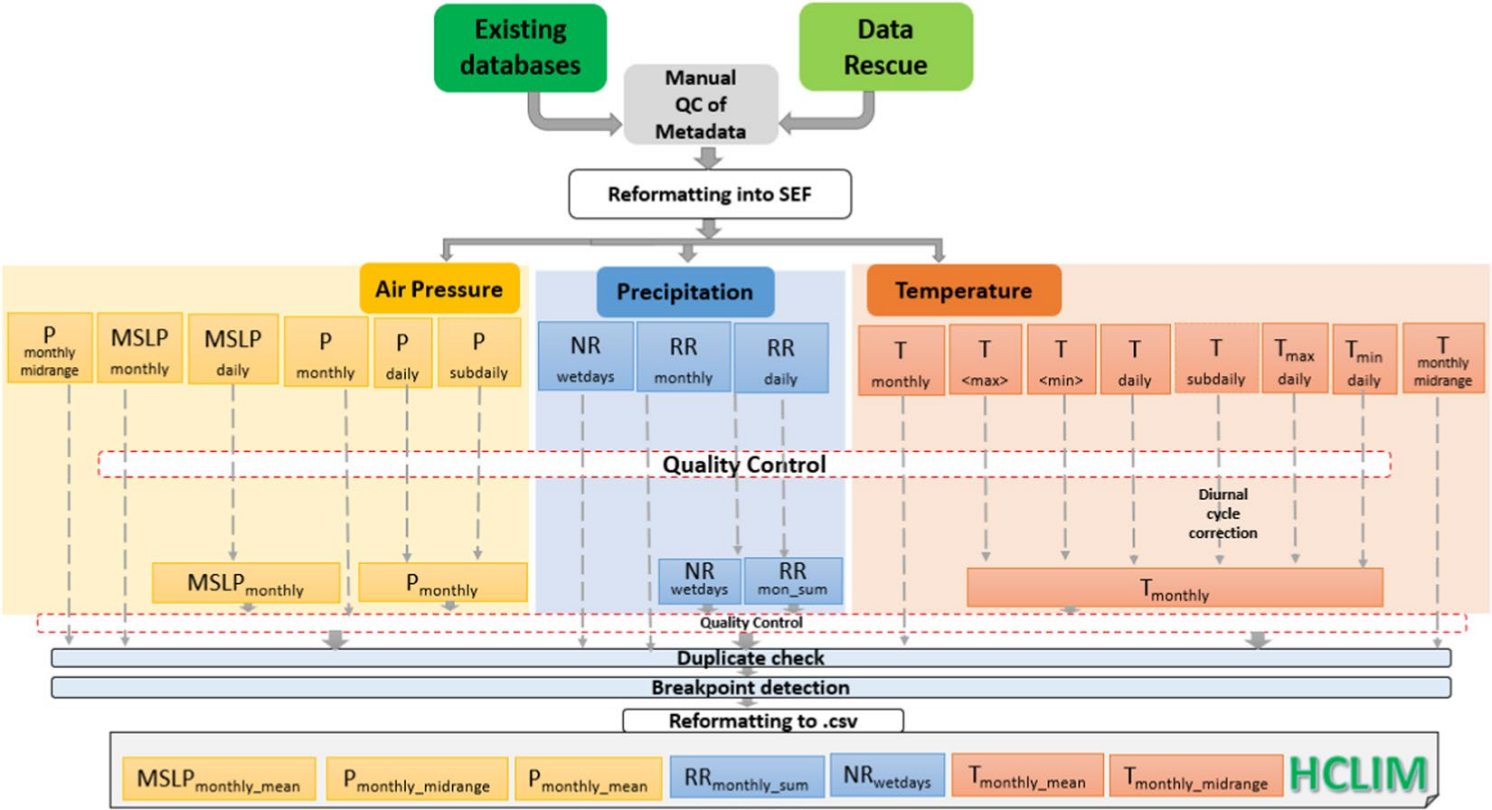
Schematic diagram, showing the sequence of steps involved in the data collection.

The processing chain is illustrated in Fig. 1. It starts with compiling data from existing databases, and rescued data. Subdaily data is processed to monthly means and then a quality control procedure (QC) is performed. Finally, the data are checked for duplicates and the detection of inhomogeneities.

### Compilation of existing data

Data were compiled from 28 available databases listed in Tables 2, 3 and 4 and in addition 16 other small data sources (Supplementary Table 1). The datasets comprise databanks with a global scope providing 27905 records to our compilation. Regional (1800), national (538) and other (61) databanks provided further records. The numbers refer to those data series compiled, i.e., only data series reaching back before 1890 that are at least one year in length. In a later process (Sect. Removing duplicates), about two thirds of the records were identified as duplicates and removed. Abbreviations given in the tables (Tables 2, 3 and 4) onwards are explained here in Table 1.

**Table 1:** Explanation of abbreviations in the Tables 2, 3 and 4.

Abbreviation	Parameter	Unit
Ta	Temperature, mean	°C (Celsius)
Tmin	Temperature, minimum	°C
Tmax	Temperature, maximum	°C
P	Air pressure, mean	hPa (Hectopascal)
MSLP	Mean sea Level Pressure	hPa
RR	Precipitation	mm (Millimetre)
NR	Number of wet days	Number

#### Global climate databases

We used six global climate databases (Table 2). They are compiled by UK and US institutions. Some of them are global temperature datasets (e.g., ISTI), whilst others have sea level pressure (e.g., ISPD) or all meteorological parameters (e.g., GHCN). Some measurements are over 300 years in age. Overview of the geographical distribution of the records can be found in Supplementary Fig. 1.

**Table 2:** Overview of Global Climate Databases.

Name	Acronyms	Spatial Coverage	Parameter (Number of records)	Resolution	Total number of records compiled	Number of records in the HCLIM dataset after removing duplicates	References	Data
Berkeley Earth Data	BEST	Global	ta (3835)	Monthly	6798	1767	^10^	^46^
			Tmax (1452)					
			Tmin (1511)					
Climatic Research Unit	CRU	Global	ta (1418)	Monthly	1418	1391	^47^	^48^
			P (83)		83	82		
Global Historical Climatology Network	GHCN	Global	ta (2870)	Monthly	3630	1049	^9^	^49^
			Tmax (372)					
			Tmin (388)					
			RR (2840)		2840	2654		
			P (24)		24	24		
A monthly global paleo-reanalysis (input)	EKF400	Global	ta (79)	Monthly	77	18	^5^	^50^
			P (101)		101	91		
			MSLP (39)		39	30		
			RR (76)		76	76		
The International Surface Pressure Databank	ISPD	Global (mostly Europe)	P (163)	Subdaily	163	118	^51^	^52^
			MSLP (258)		258	159		
International Surface Temperature Initiative	ISTI*	Global	Ta (7129)	Monthly	12398	2526	^53^	^54^
			Tmax (2634)					
			Tmin (2635)					

*Supplementary Fig. 2.

#### Regional and thematic databases

Regional and thematic databases have a narrower spatial focus (Table 3). For example, there are several sources related to the ACRE project (The international Atmospheric Circulation Reconstructions over the Earth)^16^. In addition, Supplementary Table 1 shows an overview of other smaller datasets or individual stations that were incorporated into HCLIM. Overview of the geographical distribution of these records can be found in Supplementary Fig. 3.

**Table 3:** Overview of other climate regional Databases.

Name	Acronyms	Spatial Coverage	Monthly Resolution	Daily Resolution	References	Data
			Parameter (Number of records)	Parameter (Number of records)		
Historical climate observations in Canada: 18^th^ and 19^th^ century	ACRE	Canada		Ta (25)	^55^,^56^	^57^
				Tmax (9)		
				Tmin (12)		
				P (17)		
				MSLP (17)		
ACRE South Africa	ACRE	South-Africa	Ta (8)	Ta (4)	^42^	^16^
			Tmax (6)	Tmax (5)		
			Tmin (5)	Tmin (7)		
				RR (3)		
				P (12)		
				NR (2)		
Early instrumental meteorological observations in Switzerland	CHIMES	Switzerland		Ta (66)	^58^	^20^
				Tmax (12)		
				Tmin (15)		
				RR (14)		
				P (63)		
				NR (10)		
Deutscher Wetterdienst	DWD	Overseas data	Ta (13)		^59^	^60^
			P (13)			
			RR (1)			
European Climate Assessment & Dataset	ECAD	Europe	Ta (152)		^61^	^62^
Early meteorological records from Latin-America and the Caribbean during the 18^th^ and 19^th^ centuries	EMERLAC	Latin-America and the Caribbean	Ta (18)	Ta (2)	^63^	^63^
			Tmax (11)	Tmax (1)		
			Tmin (11)	Tmin (1)		
			MSLP (4)	P (1)		
			RR (15)			
Improved Understanding of Past Climatic Variability from Early Daily European Instrumental Sources	IM-PROVE	Europe		Ta (5)	^64^	65
				P (5)		
Historical Instrumental Climatological Surface Time Series of the greater Alpine Region	HISTALP	Alps in Europe	Ta (104)		^66^	^32^
Japan-Asia Climate Data Program	JCDP	Japan	Ta (6)		^44^	^67^
			MSLP (5)			
C3-EURO4M-MEDARE Mediterranean historical climate data	MEDARE	Mediterranean region	Ta (1)		^68^	^69^
				Tmax (20)		
				Tmin (20		
				RR (20)		
				P (20)		
				NR (20)		
HARD 2.0 - Historical Arctic Database	NCU	Arctic	Ta (58)		^70^	^71^
			P (51)			
SEA-historical climate data	SEA	Australia	Ta (9)	Ta (16)	^72^	^73^
			Tmax (3)	Tmax (2)		
			Tmin (3)	Tmin (2)		
			RR (8)	RR (3)		
			MSLP (2)	MSLP (2)		
			P (12)	P (4)		
			NR (9)	NR (7)		
Southeast Asian Climate Assessment & Dataset	SACA&D	Central & South-Asia	Ta (19)		^74^	^75^
Two-Century Precipitation Dataset for the Continent of Africa		Africa	RR (233)		^11^	^76^
Forts and Volunteer Observer Database	US FORTS	United States		Ta (288)	^77^,^78^	^79^

#### National weather institutes

Most countries have their own National Weather Service. These provide weather forecasts for civilian and military purposes and conduct research in meteorology, oceanography, and climatology. Many have developed good climate databases, from which data can be extracted. Table 4 lists those that we integrated into our compilation.

**Table 4:** Overview of national weather institutions from which we have only monthly data.

Name of Institute	Acronyms	Spatial Coverage	Parameter (Number of records)	Web
Deutscher Wetterdienst	DWD	Germany	Ta (155)	^45^
Koninklijk Nederlands Meteorologisch Instituut	KNMI	Netherlands	Ta (25)	^80^
MétéoFrance	MétéoFrance	Mainland France	Ta (58)	^81^
MétéoFrance	MétéoFrance	French overseas territories	Ta (3)	^82^
			RR (39)	
Norsk Meteorologisk Institutt	MetNorway	Norway	Ta (71)	^83^
			RR (30)	
			P (16)	
Roshydromet Всероссийский научно-исследовательский институт	RIHMI-WDC	Russia	Ta (75)	^84^
Sveriges meteorologiska och hydrologiska institut	SMHI	Sweden	Ta (66)	^85^

### Data rescued

**Fig. 2:**
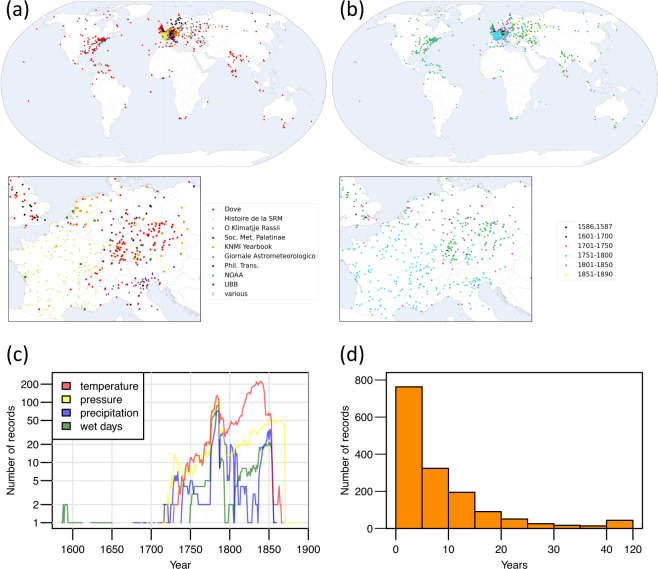
(**a**) Spatial distribution of the newly digitized records organized by source and (**b**) Start year of the rescued records. (**c**) Time evolution of the number of digitized records. (**d**) Histogram of the length of the records that have been digitized.

In addition to the data collected from various sources, we transcribed and digitized a large number of early instrumental records that were hitherto not available in digital form. Figure 2 provides maps of all the rescued records that have been digitized, categorized by sources (Fig. 2a), and start year (Fig. 2b). Figure 2c shows the time evolution of the number of digitized records and Fig. 2d shows the histogram of the length of the records that have been digitized.

The bulk of the rescued data comes from early data collections published by prominent meteorologists such as Heinrich Wilhelm Dove^17^ (1803–1879) or from early networks of weather stations such as those organized in the 18^th^ century by the Royal Society in London, Société Royale de Médecine^18^ (Royal Society of Medicine) in Paris and the Societas Meteorologica Palatina^19^ (Palatine Meteorological Society) in Mannheim. The main data sources are listed in Table 5. The reason for the peaks in the number of digitized records around 1780 and 1850 can be found in Table 5. The first peak is due to the many records belonging to the networks of the Royal Society of Medicine and the Palatine Meteorological Society); the second peak arises from the Dove^17^ and Weselowskij collections. The first peak is a bit misleading because it does not represent a large increase in spatial coverage, being most of the stations located in France and Germany. The vast majority of rescued data are short records (<20 years, see Fig. 2b) that were overlooked in previous digitization efforts.

**Table 5:** Overview of the newly digitized records.

Source	Type	Period	Variables	Resolution	Domain	# of records	# of station years
H. W. Dove^17^	Printed (periodical)	1719–1845	Ta	Monthly	Global	481	4992
Société Royale de Médecine^18^	Printed (periodical)	1776–1786	Ta, P, RR, NR	Monthly	Global	392	1642
KNMI	Printed (yearbook)	1740–1870	P	Monthly	Northern Hemisphere	60	2232
K. Weselowskij	Printed (book)	1750–1853	Ta, RR, NR	Monthly	Russian Empire	198	2028
Societas Meteorologica Palatina*^19^	Printed (yearbook)	1781–1792	Ta, P, RR, NR, others	Subdaily, Daily, Monthly	Northern Hemisphere	128	705
NOAA Climate Database Modernization Program	Handwritten	1742–1822	Ta, P, RR, NR, others	Subdaily, Monthly	United States	54	1080
G. Toaldo^86^	Printed (periodical)	1778–1810	RR	Monthly	Italy, Alps	43	426
Universitätsbibliothek Basel	Handwritten	1739–1863	Ta, P, RR, NR, others	Subdaily, Monthly	Europe	22	289
Others	Various	1586–1888	Ta, P, RR, nr, others	Subdaily, Daily, Monthly	Global	147	944

The numbers of records and station years refer to the variables included in HCLIM. A station year is defined as 12 months of non-missing data at a given station for a given variable.

*Homogenized^87^.

The inventory compiled by Brönnimann *et al*.^14^ guided us to the selection of relevant records, which needed to be easily accessible (i.e., hard copy archived in Switzerland) or already available as digital images. We digitized data from 1,235 stations corresponding to 13,822 station years for different variables at various temporal resolutions, with some duplication. The actual typing was carried out by geography students at the University of Bern.

The conversion of outside temperature and pressure to modern units followed the general procedures described in Brugnara *et al*.^20^,^21^. A few cases required additional efforts: for example, for the temperature record of Cambridge, Massachusetts (1742–1779), measured with a so-called “Hauksbee thermometer” (made for the Royal Society by Francis Hauksbee, the Younger; see^22^), we used the parallel observations with a Fahrenheit thermometer provided by the observer to build a conversion function to degrees Celsius. However, in general we discarded a large fraction of temperature records measured before ca. 1770 because of the large uncertainties in temperature scales and the lack of metadata on thermometers (these data can be obtained upon request in their original units).

We reduced pressure observations to normal gravity and, whenever possible, to 0 °C. Pressure records that were not corrected for temperature are marked by a specific metadata entry.

Some of the rescued data were already available as monthly means in existing global datasets but have been retranscribed and digitized nonetheless, to ensure a better data quality and traceability, as well as to improve daily and subdaily data availability in future projects. Some of the oldest records are calculated according to the Julian calendar or are averages of monthly extremes. These instances are flagged accordingly in the metadata.

Following best practices in data rescue (e.g.^23^), we digitized many additional variables that were observed alongside temperature and pressure. In particular: precipitation amount, precipitation type, monthly number of wet days, wind direction, wet bulb temperature, relative humidity, evaporation, snow depth, cloud cover, as well as qualitative weather descriptions. We digitized records for the number of wet days (i.e., days in which any precipitation was observed) from as early as 1586. Even though these are not strictly instrumental records, we considered them a valuable addition to the database.

The newly digitized raw data^24^ – including over 2.2 million point observations, over 120,000 daily and over 180,000 monthly statistics – have been submitted to the Global Land and Marine Observations Dataset (GLAMOD^25^^,^^26^) and will be freely available on the Copernicus Climate Change Service data store^27^. An inventory is provided in the Supplementary Information of this paper.

### From subdaily data to monthly averages

The calculation of monthly averages from subdaily observations followed two steps: 1) calculation of daily averages and 2) calculation of monthly averages from the daily averages.

To calculate daily averages, we took into account the time of observations and the effect of the diurnal cycle on averages. This is particularly important when only one observation per day is available, or when observation times are variable throughout the record.

We obtained the diurnal cycle from the nearest grid point in the ERA5-Land reanalysis, which provides hourly values of temperature and pressure since 1981 with a spatial resolution of ca. 9 km^28^. We calculated a different diurnal cycle for each calendar month from the reference period 1981–2010. To correct the raw daily means calculated from available observations, we subtracted the average of the corresponding values in the diurnal cycle, after shifting its mean to zero. For example, a daily mean obtained from a single observation in the early morning – near the time of minimum temperature – will be increased by this correction. When the observation times are not known exactly, and for stations on very small islands not resolved in ERA5-Land, the correction is not applied. A metadata entry in the monthly data files informs the user on whether the diurnal cycle correction was applied or not. For precipitation amounts, the calculation of daily values is simply the sum of all observations within a 24-hour period.

Monthly averages and sums (for precipitation) are calculated from daily values following the criteria recommended by the World Meteorological Organization^29^. The monthly average is set to missing if: (1) daily averages are missing for 11 or more days, or (2) daily averages are missing for a period of 5 or more consecutive days. Monthly precipitation sums are set to missing if any day is missing.

### Quality control

The data and metadata (geographical coordinates) in HCLIM have been quality controlled. Quality control or QC is the process to detect and label suspicious or potentially wrong values. This is necessary to avoid possible errors within datasets that could compromise the results of subsequent analysis^30^.

All metadata are deposited in a user-friendly inventory for this purpose. Information in this inventory includes station ID, name, latitude, longitude, elevation, start and end years of the time series, source, link, variable, temporal statistics (e.g., average, sum, etc.), unit (e.g., °C, mm) and other information.

The QC of the metadata in the inventory is undertaken by limit tests for latitude, longitude, and elevation, starting and ending dates of the series, variable names and units and cross-checks of the inserted country and the latitude and longitude.

For the QC of the data, we apply the following tests to each variable:

1. Range checks based on constant values. The range is shown in Table 6. This includes a check of physically impossible values such as negative values for precipitation.

**Table 6:** Upper and lower values check for the range check for different the parameters.

Parameter	Acronyms	Range
Temperature	ta, Tmax, Tmin (°C)	−60 to +40 °C
Air pressure	P (hPa)	<800 to >1050
Mean Sea Level Pressure	MSLP (hPa)	<980 to >1050
Precipitation	RR (mm, sum)	<0 to >1250
Number of rain days	NR (number)	<0 to> 28
2. Climatological outlier checks based on standard deviation, which requires at least 5 years of data. We use a threshold of 5 standard deviations.

The values that fail these tests are then confirmed manually before being flagged in the Station Exchange Format (SEF^31^) (described in the Format section under Data Records). The newly digitized data underwent additional quality checks at subdaily and daily resolution as described in Brunet *et al*.^30^.

### Removing duplicates

The next step was to create an algorithm that recognizes duplicates in the dataset. The same data can appear in several files. These can be copies of identical data compiled from several sources, different datasets (e.g., several observers in the same city), datasets supplemented with data from another city, or all possible combinations thereof (differently merged datasets). We have some examples of data from one meteorological station appearing 19 times within the 28 different databases.

The records were grouped by parameter (temperature, air pressure, precipitation, and number of wet days). Within each group we first calculated a distance (d) matrix. The second step was to calculate correlations for all pairs having d <50 km. A threshold value of >0.98 for the pair of records was set to define a duplicate. The records fulfilling both the distance and correlation criteria were then included in a merge list of the target record. Proceeding record by record, merge lists were generated for each record, and a merged record was generated according to a priority list described below. Records included in a merge were excluded from the procedure when proceeding to the next record.

Note that we did not use the station name to identify duplicates. This is because the same station might have different names, locations exist in many different languages and spelling, and different locations may have the same station name.

In each merge list, highest priority was given to the records having the earliest start date. These records were extended (or gap filled) with data series starting later. In addition, homogenized sources (of which there are few) were prioritized (e.g., HISTALP^32^). In case of identical start years (usually indicating identical data) we proceeded alphabetically. We further show an example from Madison, Wisconsin (USA) of how this method works and how the merging part of the removing duplicates takes place. Table 7 shows the merge list for this station. Note that three other stations with the name “Madison” exist (Fig. 4b) but are not in Wisconsin and represent different stations. As we do not include station names in the criteria to search duplicates, these stations were treated as separate stations as they are further away than 50 km and have a correlation below 0.98 with Madison Wisconsin.

**Table 7:** List of records from Madison.

File	HCLIM	Name	Start	End	Parameter/Resolution
1	X	USFORTS_ 470273_18530801-18830331_ta_monthly.tsv	1853	1883	ta_monthly
2	X	GHCN_v4_qcf_GHCN_USW00094811_186901-201312_ta_monthly.tsv	1869	2013	ta_monthly
3	X	GHCN_v4_qcf_GHCN_USW00014837_186901-202112_ta_monthly.tsv	1869	2021	ta_monthly
4		GHCN_v4_qcf_GHCN_USC00474966_186901-199112_ta_monthly.tsv	1869	1991	ta_monthly
5		BERKELEY_EARTH-HadleyCentreCRUpartialdatacollection_BE-68168_186901-201105_ta_monthly.tsv	1869	2011	ta_monthly
6		BERKELEY_EARTH-WorldMonthlySurfaceStationClimatology_BE-64124_186901-200412_ta_monthly.tsv	1869	2004	ta_monthly
7		ISTI-WMSSC_726410_Madison_Truax_Wis_1869_2013_ta_monthly.tsv	1869	2013	ta_monthly
8		ISTI-CRU_726410_Madison_Wisconsin_1869_2013_ta_monthly.tsv	1869	2013	ta_monthly

X represents inclusion in the HCLIM merging; no selection means they are excluded from merging to this particular Madison record in HCLIM.

**Fig. 3:**
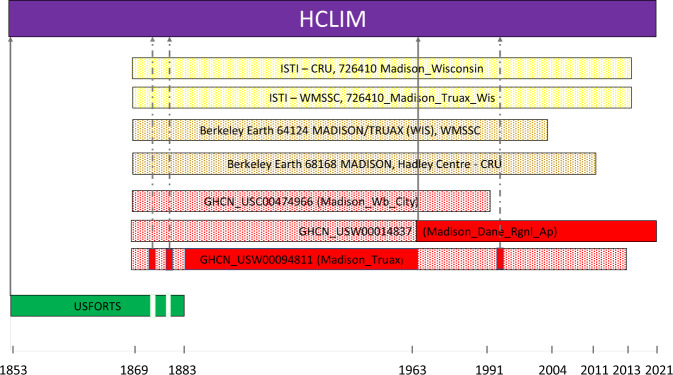
Records that were checked for duplication, deduplicated and merged from Madison.

Eight meteorological stations from Madison had temperature measurements and were tested for duplication and combined or merged, as indicated in Fig. 3.

Three stations are included in this merged Madison record. First the record from US Forts, because the record starts first or is the oldest (this is shown in Fig. 3, highlighted in green), and then two time series from GHCN (highlighted in red). The gray arrows indicate when the time series starts. Periods or observations marked with a dashed arrow show observations that are included when the time series has gaps. The Madison station (GHCN_USW00094811 Madison Truax) had some small gaps in the 1860’s and a large gap after 1963. Consequently, another GHCN record (GHCN_USW00014837 – Rgnl_Ap) becomes the dominant record through the merging process.

### Breakpoint detection

**Fig. 4:**
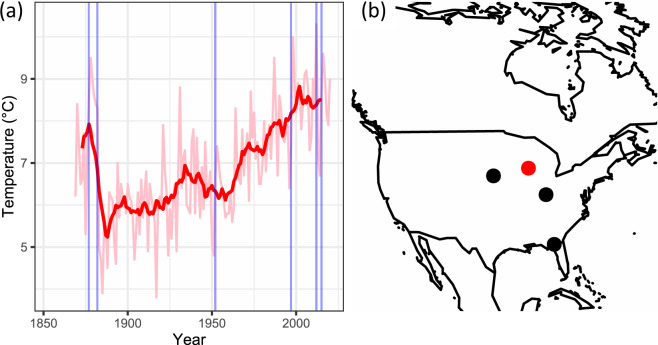
(**a**) The merged temperature record from Madison with homogeneity breaks after the breakpoint detection. The thick line shows the rolling mean or moving average, here based on every decade. (**b**) Meteorological stations named Madison in four states in the USA (Wisconsin, Nebraska, Indiana, and Florida).

The removing of duplicates causes some records to be merged, as in the example in Figs. 3,4a. This in turn can introduce large inhomogeneities in the data. We flag them using a Welch’s t-test on a 5-year moving window applied to monthly anomalies. The point in time where the inhomogeneity occurs, or breakpoint, is where the maximum of the absolute value of the test statistic occurs. The procedure is similar to the Standard Normal Homogeneity Test^33^, but we require the size of the inhomogeneity to be larger than the average of the standard deviations of the two data segments that are separated by the breakpoint. In addition, we consider data gaps of 10 years or longer as breakpoints independently of the results of the statistical test.

Figure 4a shows the merged temperature record from Madison (1853–2021) with 6 breakpoints found through breakpoint detection (1877, 1882, 1952, 1997, 2012 and 2015). Only one of the breakpoints (1882) corresponds to a merging point. It is associated with a large step inhomogeneity. We did not homogenize data in HCLIM, but we provide the breakpoints and the merging information, both of which can be used for homogenization.

## Data Records

### Overview

After eliminating obvious duplicates and applying the ‘removing duplicates’ algorithm, we ended up with 12452 merged meteorological time series across 4 parameters. These series constitute the HCLIM dataset.

The HCLIM dataset has been deposited in a public repository and can be easily downloaded from the site^15^. Table 8 provides an overview of the numbers of records downloaded for the various parameters in each step of the data processing in the HCLIM dataset.

**Table 8:** Overview of the number of records through the development of HCLIM from downloading and digitization to HCLIM dataset.

Parameter	Total downloaded	Unique records before merging (original data)	After merging => HCLIM dataset
Temperature (Ta)	17228	7573	3632
Air pressure (P)	827	827	806
Mean Sea Level Pressure (MSLP)	317	317	
Precipitation (RR)	6378	6378	4943
Number of rain days (NR)	3155	3155	3071
Sum	27905	18250	12452

Table 9 indicates how many years and stations years are included in the HCLIM dataset. In total there are over one million station years, of which 148,843 are before 1891 (Table 8). The largest numbers, both in terms of stations years and number of stations, concern precipitation (Table 8). The variable with the least number of stations and station years is pressure.

**Table 9:** Overview of number of station years and records after the removing duplicates.

Parameter	Station years total	Stations years before 1891	Number of stations
Temperature	328,794	56,384	3632
Pressure	33,264	15,406	806
Precipitation	441,701	57,288	4943
Wet days	215,692	19,931	3071

### Format

**Fig. 5:**
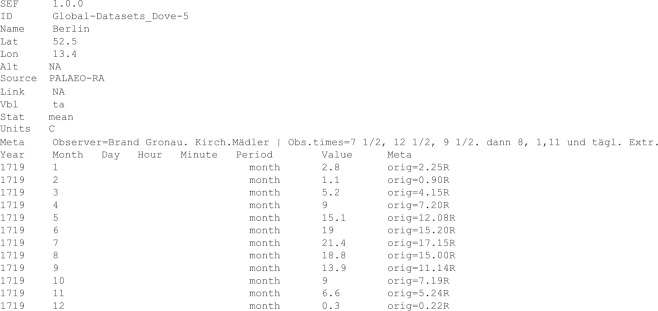
Example of a SEF file: Temperature record from Berlin 1719, from the Dove Collection.

All data were reformatted to the Station Exchange Format (SEF). This is a format introduced by the Copernicus Climate Change Service^31^. It provides a simple but standard format for the distribution of historical weather data. SEF files have a.tsv format (tab-separated values) and list basic metadata regarding the station and the data manipulation in a header. The SEF is designed for rescuing observations and present them for widespread use in an uncomplicated format and made accessible through publicly available software. The aim of such SEF files is that they can be easily integrated into global repositories^31^. An example is shown in Fig. 5. The data are also available in a single compact flat file (.csv format) where, however, no metadata are provided. Geographical coordinates can be retrieved from an inventory file.

### Temporal and spatial coverage

All the earliest meteorological records started in Europe. The first record is for wet days in Resterhafe-Osteel (Germany), which started in 1586. The first instrumental records started in 1658 for temperature, 1670 for air pressure and 1688 for precipitation; all of which are from Paris. Table 10 lists the oldest records in HCLIM, organized continentally. For example, the first thus far known temperature measurements outside Europe are for Charleston in the USA, beginning in 1738.

**Table 10:** First and oldest meteorological observations/measurements organized according to continents and parameters in HCLIM.

	Temperature	Air pressure	Precipitation	Number of rain days
Europe	Paris (1658)	Paris (1670)	Paris (1688)	Resterhafe-Osteel (1586)
Africa	Funchal (1747)	Funchal (1747)	Funchal (1747)	Freetown (1793)
Asia	Chandannagar (1741)	Beijing (1757)	Beijing (1757)	Beijing (1757)
North America	Charleston (1738)	Toronto (1734)	Cambridge (1742)	Charleston (1738)
Central- and South America	Westmoreland, Jamaica (1764)	Rio de Janeiro (1795)	Westmoreland, Jamaica (1760)	Mexico City (1775)
Australia	Sydney (1788)	Sydney (1788)	Paramatta (1822)	Sydney (1788)

The start year is given in parenthesis.

**Fig. 6:**
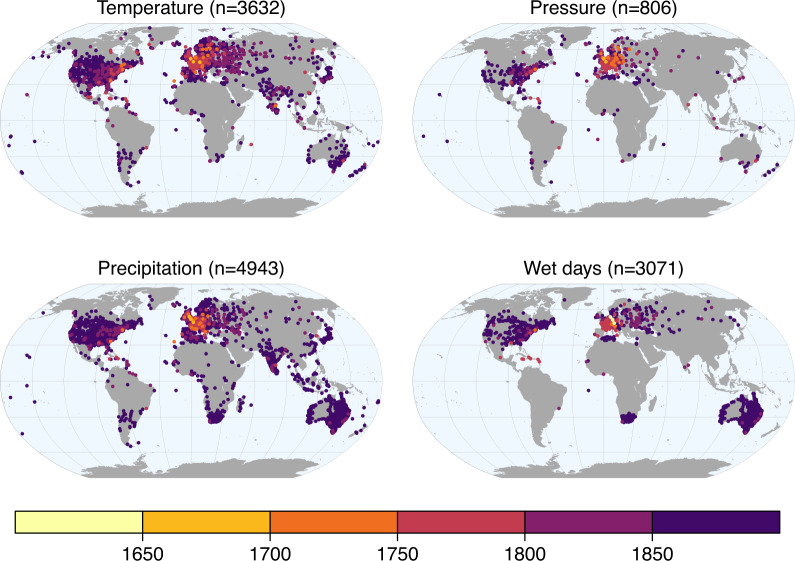
Temperature, pressure, precipitation, and wet day records in HCLIM by start year.

**Fig. 7:**
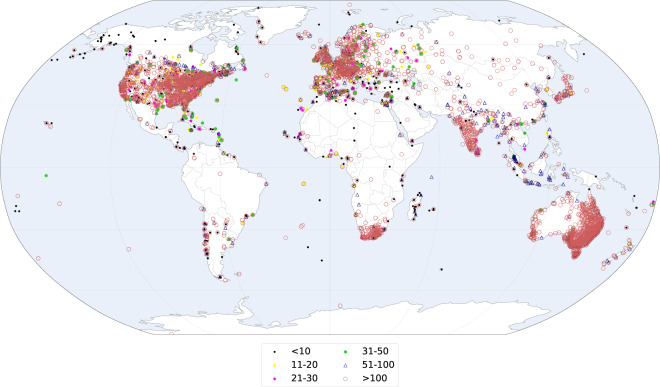
Length of the time series in years before 1891.

Figures 6, 7 show global maps of the available records sorted by start year and length.

Our collection includes 2359 temperature records, 3134 precipitation records, 160 air pressure records and 1551 number of wet days records that contain more than 100 years of data. For temperature 117 records contain more than 200 years and 5 records more than 300 years. The five longest records are listed in Table 11.

**Table 11:** Overview of the five longest weather records per parameter before 1891.

Temperature (Ta)	Precipitation (RR)	Air Pressure (P/MSLP)	Wet Days (NR)
Paris (233)	Paris (203)	Paris (221)	Padova (291)
CET, London (232)	Kew, London (194)	London (199)	Hoheissenberg (238)
Berlin (190)	Zürich (183)	Zürich (183)	Palermo (211)
DeBilt (185)	Padova (166)	Vienna (182)	Praha-Klementinum (201)
Padova (165)	PodeHole-3, Spalding (165)	Prešov (174)	Bologna (194)

The length in years is given in parenthesis, the operating years is calculated into years affected (missing months may occur).

Outside Europe, the Boston record is the 11^th^ longest for temperature with 148 observation years before 1891. Westmoreland at Jamaica, situated in the Caribbean Sea in Central America has the longest time series for precipitation record with 131 years of observations before 1891, having started in 1760, and is the 33^rd^ longest in HCLIM. Adelaide in Australia has the longest record outside Europe for number of rain days, parameter with 103 years before 1891. Precipitation had better coverage in Africa and Australia, pressure is even more limited to Europe.

**Fig. 8:**
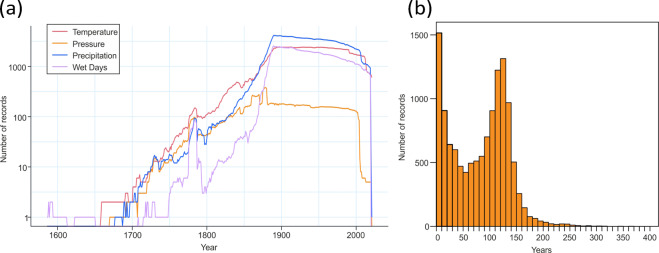
(**a**) Time evolution of the number of records per parameter (note the logarithmic scale). (**b**) Histogram of the length of all the records until 2021.

Figure 8 provides an overview of the compilation of all records in HCLIM until 2021. Figure 8a shows the start years and the distribution per parameter, while Fig. 8b shows the length of the records. The maximum length of ~150 years is largely a product of not having considered series starting after 1890.

As seen in Fig. 8a, the typical trend is that most records began in the late 19^th^ century round year 1880–90 (pressure is little earlier) and it increases and ends up with an explosive development towards the end of the seventeenth century for all parameters.

However, the largest increase in the number of meteorological records occurred in the mid and late 19^th^ century. This development does not apply to air pressure data, mostly because not as many data rescue projects have targeted this variable.

### Examples

The era of the first recorded meteorological observations begins during the 17^th^ century in Europe. Here we show a few examples of the earliest and longest time series for each parameter.

**Fig. 9:**
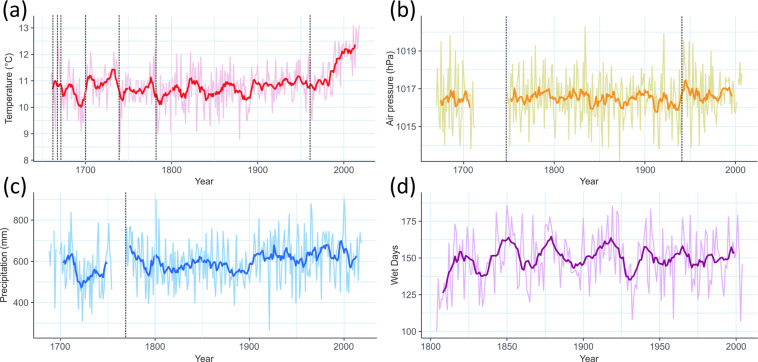
(**a**) The oldest and longest temperature time series in the world from Paris that shows mean annual temperature for the years 1658–2018. The black dashed lines indicate homogeneity breaks (HCLIM)^15^. (**b**) The oldest and longest air pressure time series in the world from Paris, showing mean annual air pressure in hPa for the years 1670–2007 (the time series has a gap between 1726–1747). (**c**) The oldest and longest precipitation annual record from Paris (1688–2018). (**d**) The oldest and longest annual record for number of rain days from Prague^88–103^.

Paris has the earliest and longest meteorological records lasting over 200 years for three meteorological parameters (temperature, pressure, and precipitation). Rousseau^34^,^35^ developed a monthly temperature record available back to 1658. This is the longest continuous instrumental meteorological record (duration: 360 years). Figure 9a shows the annual temperature time series from Paris, with breakpoints marked. Annual averages are calculated as the average or the sum, it depends on the parameter (WMO^36^), and if there are missing values, the additional uncertainty introduced in the estimation of an average monthly value described in WMO^29^, for example, is also taken into account.

After Evangelista Torricelli’s invention of the barometer in 1643, systematic measurements of pressure began in 1670, also in Paris. From this, the Paris MSLP (Mean Sea-Level Pressure) record was compiled and published by Cornes *et al*.^37^. Unfortunately, a gap in the series still exists for the period 1726–1747, for which it seems that no barometer observations have survived, however, there is no clear inhomogeneity in the longer dataset. The annual average MSLP for Paris (1670–2007) is presented in Fig. 9b.

The Paris precipitation record (Fig. 9c) begins in 1688 and is the longest known continuous precipitation record^38^. There is a long gap in the record (18 years after 1755), to which a breakpoint is assigned by definition.

Prague has the longest time series (Fig. 9d) for rain days without any gap.

## Technical Validation

As described in the Methods section, we provide raw data. Result from the QC control provides an indication of the general data quality (Table 12). Most QC flags are for precipitation. The well-known global databases have few QC flags (Table 12). This became clear when every single database that was downloaded was quality controlled. These are probably previously well controlled. It should also be mentioned that until the end of the 19^th^ century there was no standard regulation for meteorological observations^16^,^39^. However, to be precise the first international standards were set in 1873, although it was mostly about observation times and reporting standards^40^.

**Table 12:** Overview of the percentage of flagged data for the quality control in HCLIM.

Source	Period	Variables	Fraction of flags after quality control	
			1658–1890 (232 years)	1891–2021 (130 years)
HCLIM-Dataset	1658–2019	Ta	0.0066%	0.022%
		P	0.27%	0.015%
		RR	0.4%	0.41%
		NR	0.098%	0.12%

For these reasons, testing the raw data and all the available metadata is the best option with which to optimize the use of the database. Every individual user will be able to apply the type of post-processing that is best suited to their needs.

After applying our breakpoint detection algorithm, we find large inhomogeneities in ca. 76% of the temperature records, 41% of the pressure records, 48% of the precipitation records, and 77% of the wet days’ records (after de-duplication). This corresponds to an average homogeneous period (i.e., number of station years divided by the number of breakpoints) of 33, 56, 129, and 37 years, respectively.

We stress that smaller inhomogeneities remain undetected and that the detection is less effective for precipitation series, where the signal-to-noise ratio is very low. This can be relevant for applications such as trend analysis and would require more advanced detection methods that make use of reference series.

## Usage Notes

The data products can be widely used in climate change research, such as reconstructions and data assimilations. Our database is based on an equivalent methodology that was previously developed by many others (GHCN, ISTI, Berkeley Earth etc). But this product represents the most comprehensive pre-industrial global dataset at a monthly temporal resolution. The data have been quality controlled and duplicate dataset removed. Although a breakpoint detection has been performed, complete homogenization is still required. Hence, we do not recommend using the dataset for trend analyses at the current stage, but the utility of the database is equally valuable, for analysis of singular extreme events, or the impact of volcanic eruptions (Laki in 1783, Tambora in 1815 and the Year Without a Summer, etc).

## Supplementary information

Supplementary Figure 1

Supplementary Figure 2

Supplementary Figure 3

Supplementary information

## Data Availability

R code used for formatting, quality control, removing duplicates, and breakpoint detection are publicly available under https://github.com/elinlun/Hclim. The data are available at PANGAEA^15^.
