# Supplementary figures and images for: The global historical climate database HCLIM
Source: Sci Data. 2023 Jan 19;10:44. doi: 10.1038/s41597-022-01919-w (PMC9851593; doi:10.1038/s41597-022-01919-w)

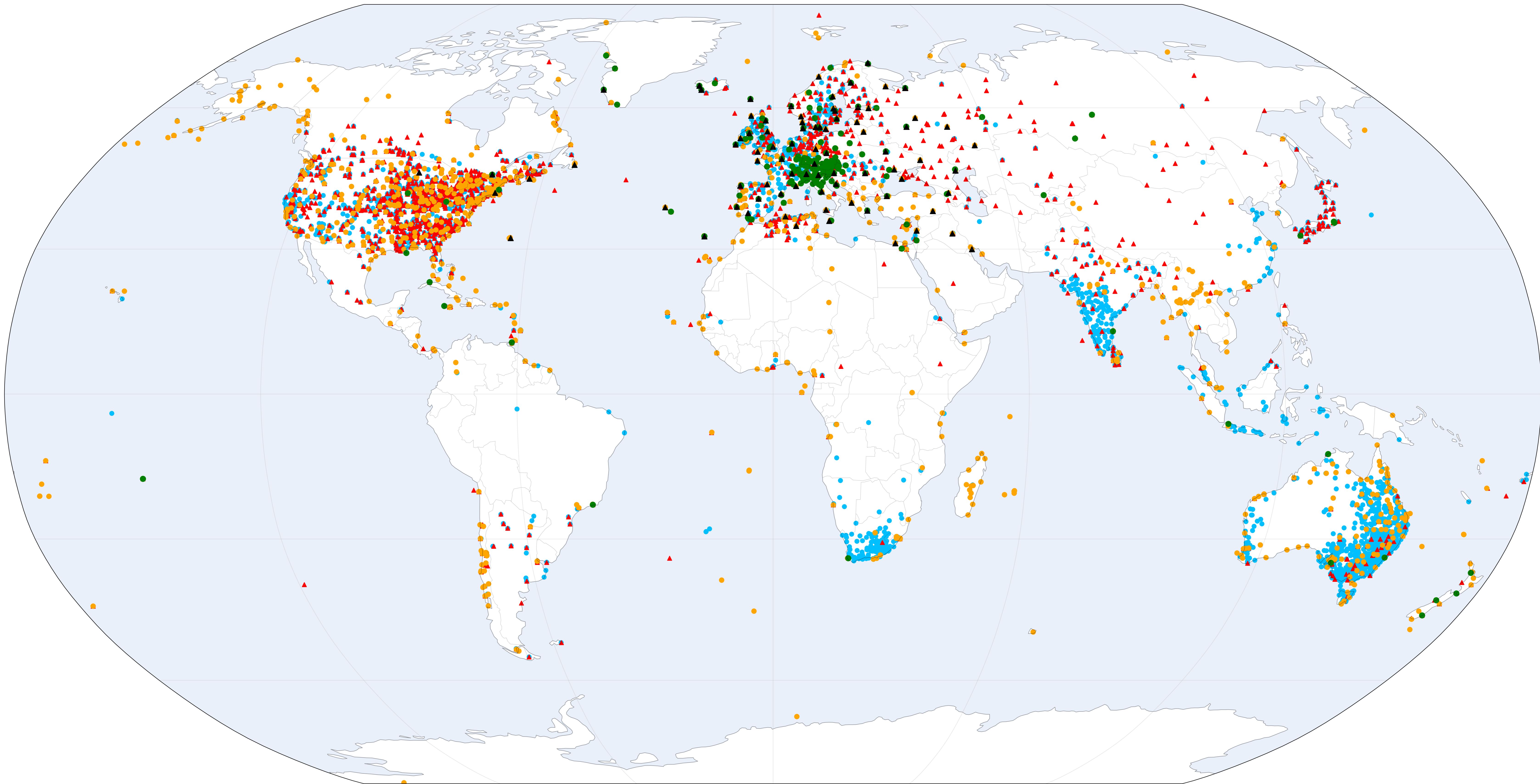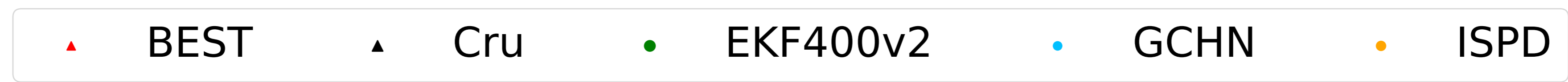

Supplement: Supplementary file 1 — Supplementary Figure 1 [file 41597_2022_1919_MOESM1_ESM.pdf]

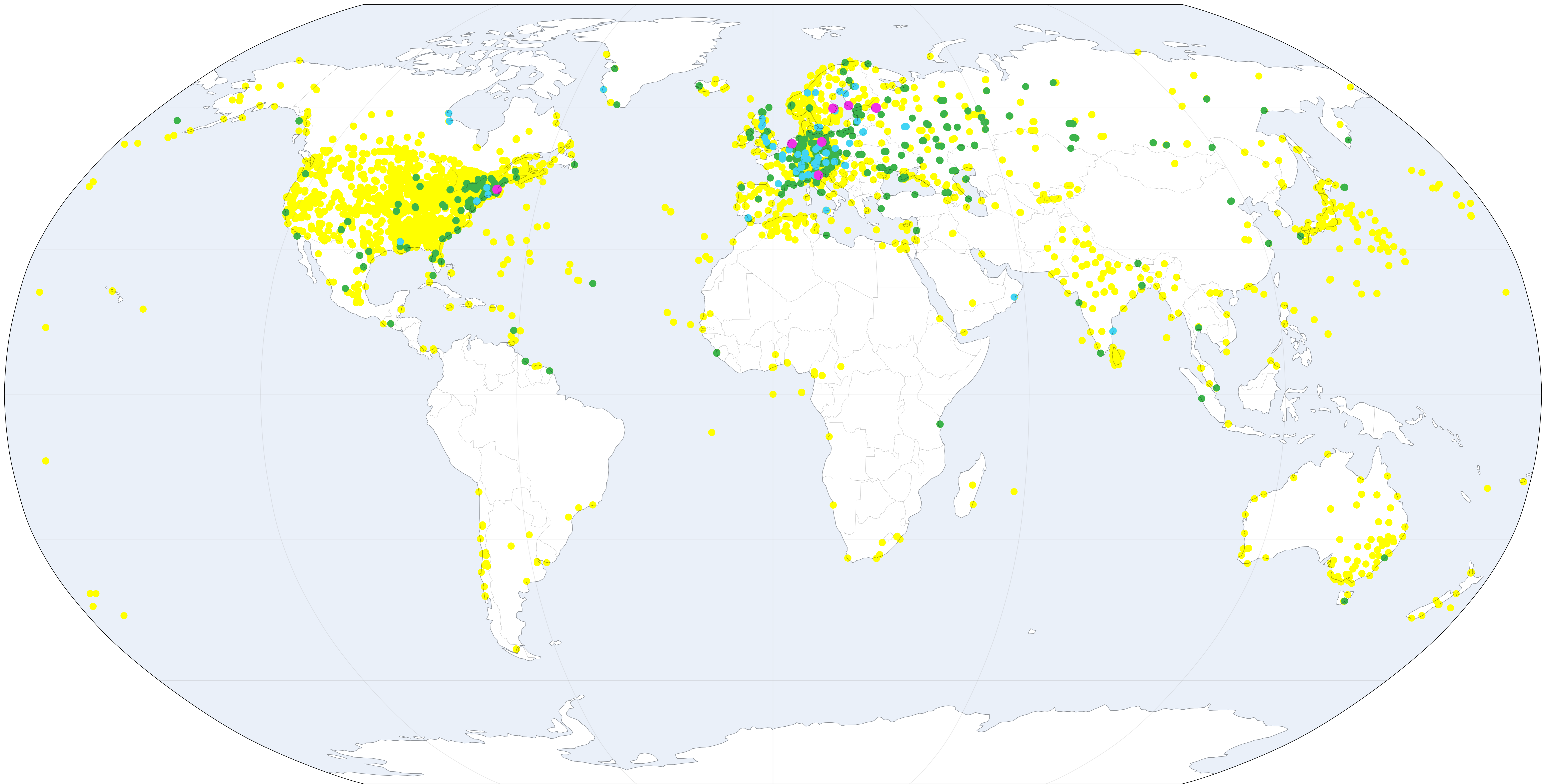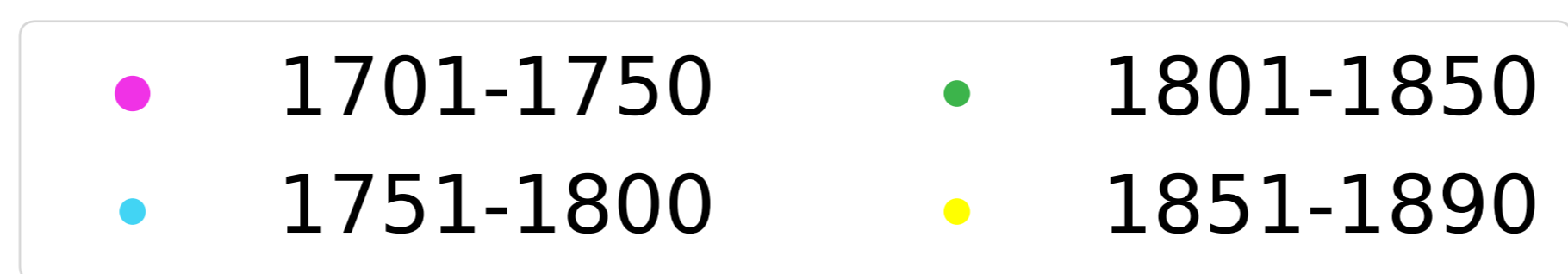

Supplement: Supplementary file 2 — Supplementary Figure 2 [file 41597_2022_1919_MOESM2_ESM.pdf]
