# Supplementary material for: The global historical climate database HCLIM
Source: Sci Data. 2023 Jan 19;10:44. doi: 10.1038/s41597-022-01919-w (PMC9851593; doi:10.1038/s41597-022-01919-w)
Supplement: Supplementary file 3 — Supplementary Figure 3 [file 41597_2022_1919_MOESM3_ESM.pdf]

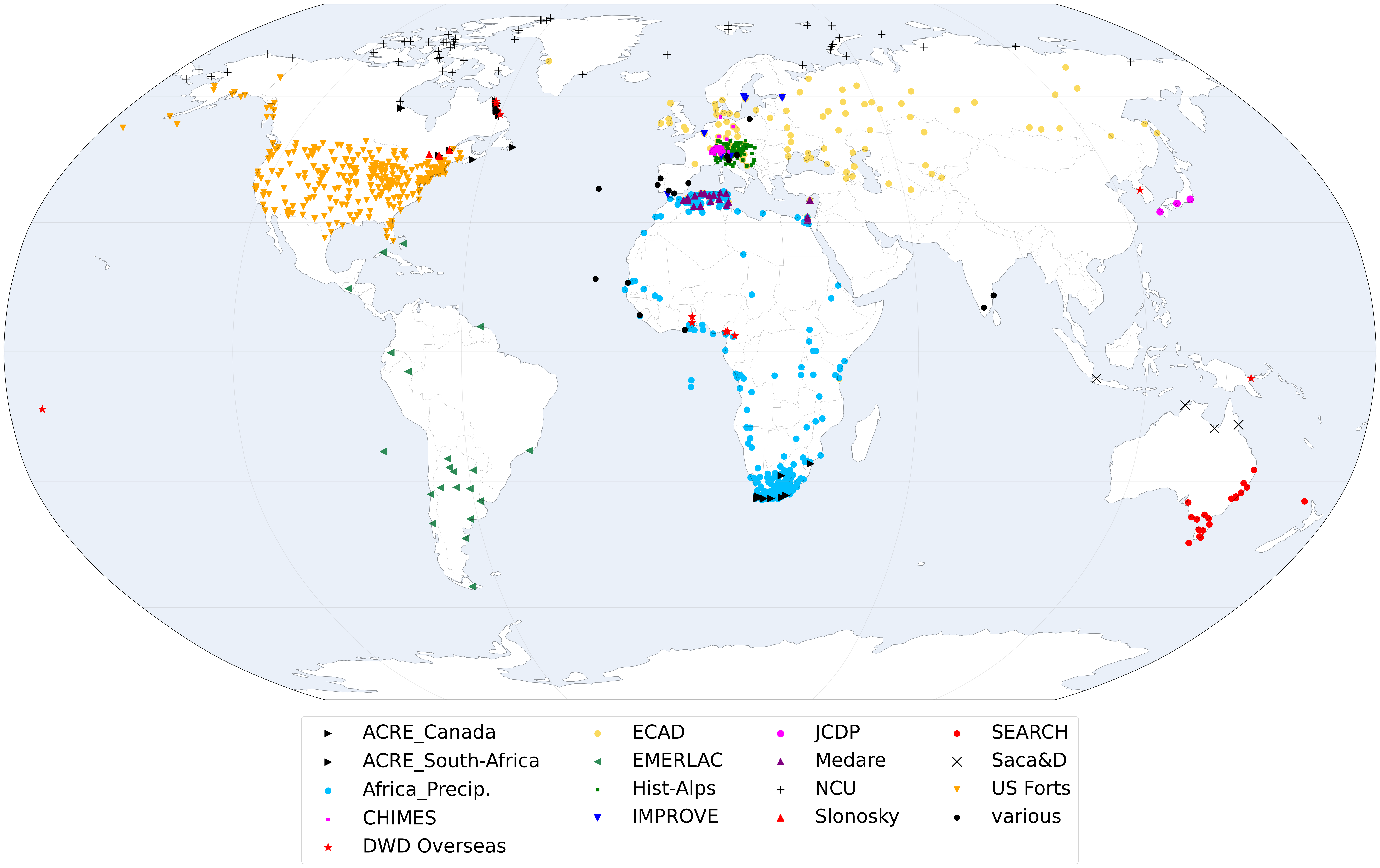

ACRE_Canada
ACRE_South-Africa
Africa_Precip.
CHIMES
DWD Overseas
ECAD
EMERLAC
Hist-Alps
IMPROVE
JCDP
Medare
NCU
Slonosky
SEARCH
Saca&D
US Forts
various
