# Supplementary material for: The global historical climate database HCLIM
Source: Sci Data. 2023 Jan 19;10:44. doi: 10.1038/s41597-022-01919-w (PMC9851593; doi:10.1038/s41597-022-01919-w)
Supplement: Supplementary file 4 — Supplementary information [file 41597_2022_1919_MOESM4_ESM.pdf]

Supplementary information

Table of content

Supplementary Table 1. Other small datasets or individual stations..........................**page 1**

Supplementary Figure 1. Temperature records from the global databases shown with different sources... **page 2**

Supplementary Figure 2. Stations from the ISTI dataset... **page 2**

Supplementary Figure 3. Stations from the regional databases categorized according to sources.. **page 3**

Supplementary Table 1. *Other small datasets or individual stations*

References	Spatial Coverage	Monthly Resolution Parameter (Number of records)	Daily Resolution Parameter (Number of records)	Name	Web address and data
Allan et al., 2011	Chile<br>Iceland		Ta (5)<br>P (6)	ACRE	25 & 29
Alcoforado et al., 2012	Portugal	Ta (2)<br>RR (2)<br>NR (2)		Early Portuguese Meteorological Measurements	88
Brázdil et al., 2008	Slovakia		Ta (1)<br>P (1)	Eperjes<br>(Now: Prešov)	89
Kunz et al., 2022	Karlsruhe, Germany		T (1)<br>P (1)	The Karlsruhe climate time series since 1779	90
Camuffo, et al., 2020	Italy		RR (1)	Three centuries of daily precipitation in Padua, Italy	91
Camuffo, et al., 2020	Italy		Ta (1)	Temperature observations in Florence, Italy, after the end of the Medici Network	92
Camuffo, et al., 2017	Italy		Ta (1)	Temperature observations in Bologna, Italy	93
Cornes, R.C., 2020	England	Ta (1)<br>P (1)	Ta (1)<br>P (1)	Robert Boyle's weather journal for the year 1685	94
Demarée, G.R. & Ogilvie, A.E.J., 2011	Labrador, Canada	Ta (1)<br>P (1)		The Case of Iceland and Labrador	95
Dominguez-Castro, et al., 2014	Spain	RR (1)<br>NR (1)		Early Spanish meteorological records	96
Filipiak, 2010	Poland	Ta (1)<br>RR (1)		History of the Gdańsk Pre-Instrumental	97
Rodrigo, F.S., 2019	Spain, West-Africa	Ta (2)<br>P (1)<br>RR (2)<br>NR (3)		The climate of Granada (southern Spain) & EMOWA, Early Meteorological Observations in West Africa	98 & 99
Slonosky, V. C., 2015	Canada		Ta (16)	Daily minimum and maximum temperature in the St-Lawrence Valley, Quebec	28 & 100<br>(Part of ACRE_Canada)
Slonosky, V. C., 2003	Quebec, Canada		Ta (1)	The Meteorological Observations of Jean-François Gaultier, Quebec, Canada:	101
Thomas Jefferson's weather journal	USA<br>France		Ta (4)<br>P (1)	The Jefferson Weather & Climate Record	102
Ives, G., 2020	India	RR (3)		A history of the monsoon in southern India between 1730 and 1920 and its impact on society	103

The supplementary figures show originally global databases such as for instance the Berkely Earth database (BEST) in Figure 1. Figure 2 shows the ISTI dataset. Supplementary Figure 3 shows regional datasets.

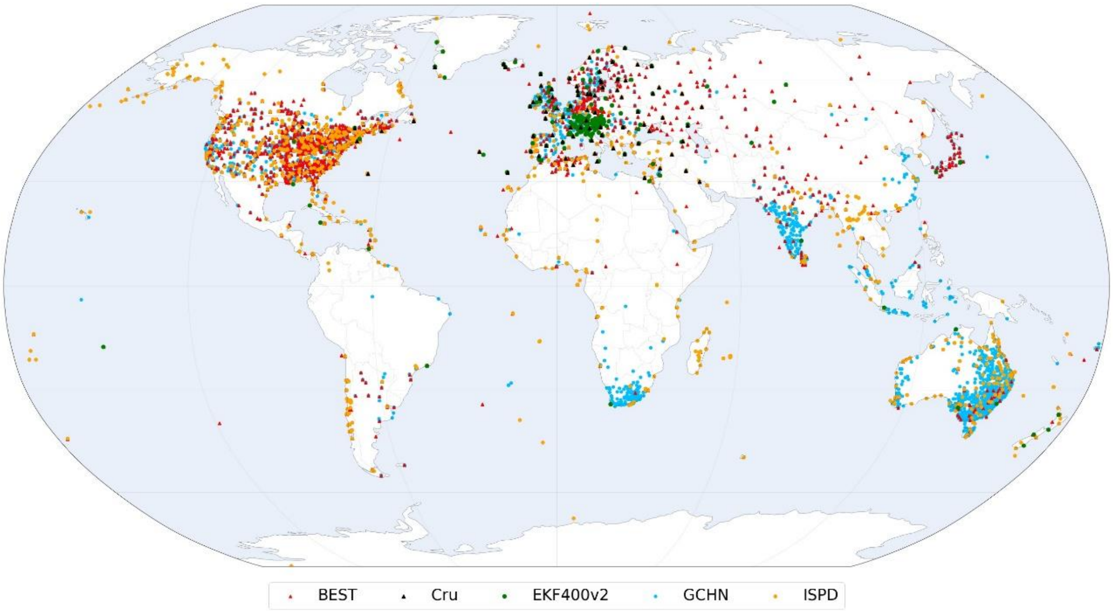

Supplementary Figure 1. Temperature records from the global databases shown with different sources.

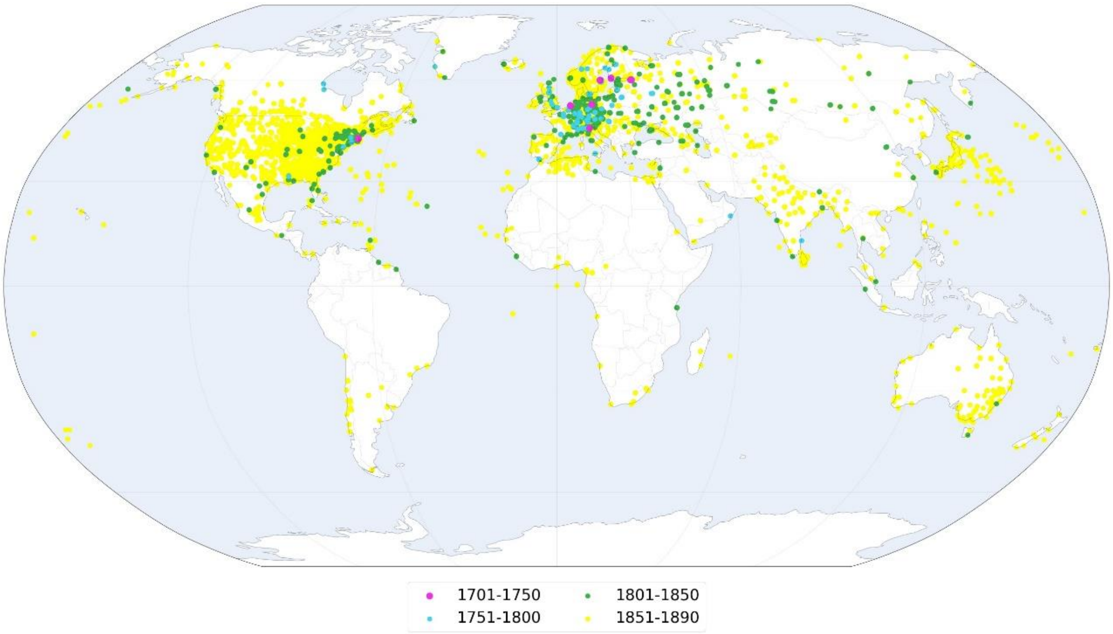

Supplementary Figure 2. Stations from the ISTI dataset.

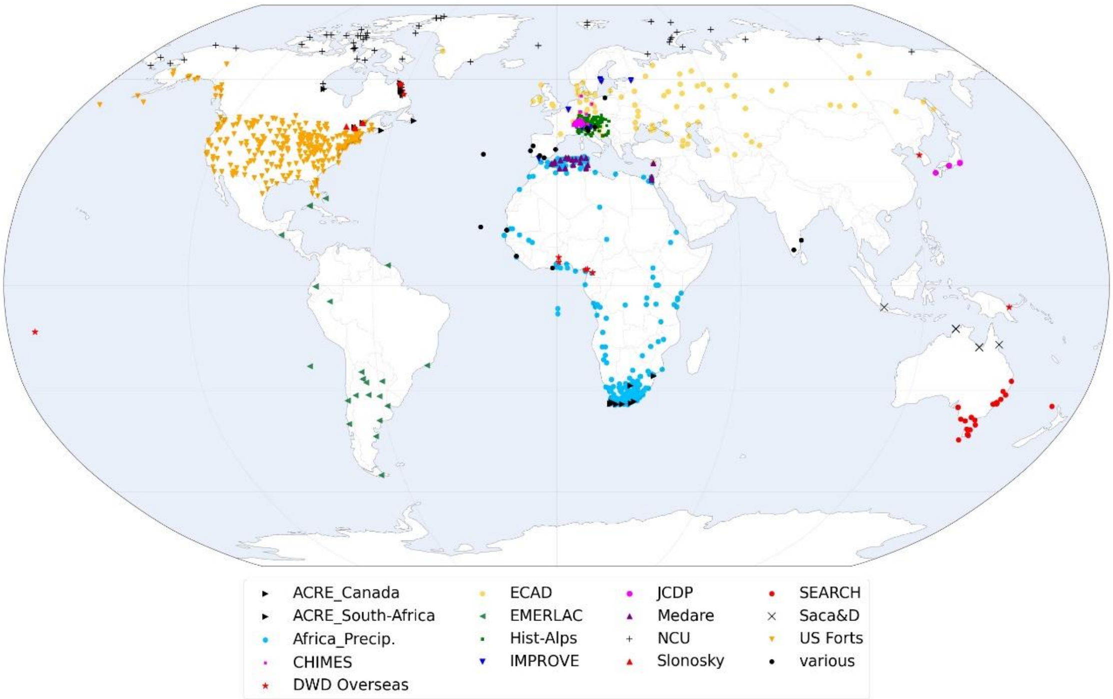

Supplementary Figure 3. Stations from the regional databases categorized according to sources.
